# Ensuring the Robustness and Reliability of Data-Driven Knowledge Discovery Models in Production and Manufacturing

**DOI:** 10.3389/frai.2021.576892

**Published:** 2021-06-14

**Authors:** Shailesh Tripathi, David Muhr, Manuel Brunner, Herbert Jodlbauer, Matthias Dehmer, Frank Emmert-Streib

**Affiliations:** ^1^ Production and Operations Management, University of Applied Sciences Upper Austria, Linz, Austria; ^2^ Department of Computer Science, Swiss Distance University of Applied Sciences, Brig, Switzerland; ^3^ School of Science, Xian Technological University, Xian, China; ^4^ Department of Biomedical Computer Science and Mechatronics, UMIT-The Health and Life Science University, Hall in Tyrol, Austria; ^5^ College of Artificial Intelligence, Nankai University, Tianjin, China; ^6^ Predictive Society and Data Analytics Lab, Faculty of Information Technology and Communication Sciences, Tampere University, Tampere, Finland; ^7^ Institute of Biosciences and Medical Technology, Tampere University, Tampere, Finland

**Keywords:** machine learning, robustness, industry 4.0, smart manufacturing, industrial production, CRISP- DM

## Abstract

The Cross-Industry Standard Process for Data Mining (CRISP-DM) is a widely accepted framework in production and manufacturing. This data-driven knowledge discovery framework provides an orderly partition of the often complex data mining processes to ensure a practical implementation of data analytics and machine learning models. However, the practical application of robust industry-specific data-driven knowledge discovery models faces multiple data- and model development-related issues. These issues need to be carefully addressed by allowing a flexible, customized and industry-specific knowledge discovery framework. For this reason, extensions of CRISP-DM are needed. In this paper, we provide a detailed review of CRISP-DM and summarize extensions of this model into a novel framework we call Generalized Cross-Industry Standard Process for Data Science (GCRISP-DS). This framework is designed to allow dynamic interactions between different phases to adequately address data- and model-related issues for achieving robustness. Furthermore, it emphasizes also the need for a detailed business understanding and the interdependencies with the developed models and data quality for fulfilling higher business objectives. Overall, such a customizable GCRISP-DS framework provides an enhancement for model improvements and reusability by minimizing robustness-issues.

## Introduction

Since the beginning of industry 4.0 initiatives, the concept of smart manufacturing has gained considerable attention among researchers from academia and industry. Specifically, data-driven knowledge discovery models are now regarded as an essential pillar for smart manufacturing. The concept of intelligent manufacturing systems was initially discussed in Refs. (Hatvany and Nemes, 1978; Hatvany and Lettner, 1983), where the authors addressed the prospects of systems and their complexities and emphasized that systems should be built to be resilient to unforeseen situations and to predict trends in real time for large amounts of data.

In recent years, the idea of smart manufacturing developed further using the framework of multi-agent systems (MASs). MASs are groups of independent agents that cooperate with each other and are capable of perceiving, communicating, reproducing, and working not only toward a common goal but also toward individual objectives. An agent is composed of several modules that enable it to work effectively both individually and collectively. The acting module of a learning agent collects data and information (percepts) from the external world through sensors and responds through effectors, which results in actions. The learning module and critical module of an agent react to improve the actions and the performance standards by interacting with each other. Furthermore, the problem generator module enforces the exploratory efforts of the agent to develop a more appropriate world view (Russell and Peter, 1995; Khalid et al., 1997; Monostori, 2003; Lee and Kim, 2008; Wang et al., 2016).

The learning agent is a software program or an algorithm that leads to an optimal solution of a problem. The learning processes can be classified into three categories: 1) supervised, 2) unsupervised, and 3) reinforcement learning. The learning module is a key driver of a learning agent and puts forward a comprehensive automated smart manufacturing process for autonomous functioning, collaboration, cooperation, and decision making. The availability and potential of data from an integrated business environment allow various business objectives to be formulated, such as automated internal functioning, organizational goals, and social and economic benefits. Various business analytic methods, metrics, and machine learning (ML) strategies serve to analyze these business objectives. For instance, Gunther et al. (Mohammad, 2017) extensively reviewed big data in terms of their importance for social and economic benefits. Furthermore, they highlighted three main issues of big data in order to realize their potential and to match up with the ground realities of business functioning (i.e., how to transform data into information to achieve higher business goals). The three issues considered are work-practice goals, organizational goals, and supra-organizational goals. The availability of big data allows us to apply various complex ML models to various manufacturing problems that could not be addressed previously. Ross et al. (2013) discussed the big data obsession of business organizations rather than asking the straightforward question of whether big data are useful and whether they increase the value of business-related goals. They concluded that many business applications might not need big data to add value to business outcomes but instead need to address other organizational issues and business rules for evidence-based decision making founded on rather small data. However, their paper does not discuss manufacturing and production but instead focuses on issues related to business management. Nevertheless, a similar understanding may be obtained in many other production- and manufacturing-related instances that do not require big production data but instead require sufficient samples of observational or experimental data to support robust data analytics and predictive models.

Kusiak (2017) discusses key features of smart manufacturing and considers predictive engineering as one of the essential pillars of smart manufacturing, and (Stanula et al., 2018) discuss various guidelines to follow for data selection for understanding business and data in manufacturing. Wuest et al. (2014), Wuest et al. (2016) discuss the various challenges encountered in ML applications in manufacturing, such as data preprocessing, sustainability, selection of the appropriate ML algorithm, interpretation of results, evaluation, complexity, and high dimensionality. The challenges raised by Wuest et al. (2014), Wuest et al. (2016) requires a systematic and robust implementation of each phase as defined via the Cross-Industry Standard Process for Data Mining (CRISP-DM) framework in a manufacturing environment. Kusiak (Kristoffersen et al., 2019) discusses the five key points that highlight the gaps in innovation and obstruct the advancement in smart manufacturing and emphasizes that academic research must collaborate extensively to solve complex industrial problems. The second point is about new and improved processes of data collection, processing, regular summarization, and sharing. The third point is to develop predictive models for outcome prediction. The fourth point deals with developing general predictive models to capture trends and patterns in the data to overcome future challenges instead of memorizing data by feeding in large amounts. The fifth point is to connect factories and processes that work together by using open interfaces and universal standards. Kusiak (Kristoffersen et al., 2019) also emphasizes that smart manufacturing systems should adopt new strategies to measure useful parameters in manufacturing processes to match up with new requirements and standards. The studies discussed above emphasize the development of ML models and their robustness so that ML can effectively meet the new manufacturing challenges. These robustness issues may be attributed to faulty sensors, corrupt data, missing data or data drifting.

The papers discussed above highlight the importance of big data in smart manufacturing and production. These studies emphasized various issues related to robust ML prediction models and guidelines for systematic CRISP-DM model implementation and achieving business goals utilizing big data. However, these studies addressed these issues largely in isolation and did not consider, e.g., systematic implementation of different phases of data and modeling related issues considering CRISP-DM and interactions between a data-driven Knowledge discovery model framework (CRISP-DM) and machine learning models. Neither have model transparency issues been considered in sufficient depth.

The challenges at different phases of CRISP-DM are interrelated for a data-driven model from the beginning of the business understanding that formulates business hypotheses and goals to the data understanding and preparation, modeling and model deployment that validates the business hypotheses. It is required that the business goals and data-characteristics, models’ attributes and characteristics and users’ understanding must be quantitatively or qualitatively recorded and evaluated. Each phase of CRISP-DM shows sensitivity towards internal parameters and the external parameters (that is caused by the other phases). Therefore a robust CRISP-DM must consider the hypothesis, which tests the influence between phases, i.e.,

$$\begin{array}{l} H_{0}:\rho \left(a,b|B,D_{x},M_{a},M_{b},U_{x},T_{i}\right)=\rho \left(a,b|B,D_{y},M_{a},M_{b},U_{y},T_{j}\right)\\ H_{1}:\rho \left(a,b|B,D_{x},M_{a},M_{b},U_{x},T_{i}\right)\neq \rho \left(a,b|B,D_{y},M_{a},M_{b},U_{y},T_{j}\right) \end{array}$$ (1)

Here, *ρ*(a,b) measures the influence between two phases of CRISP-DM *a* to *b*, *B* is a business goal, *Dx* and *Dy* are the two different data-sets at timepoint *T*_*i*_ and *T*_*j*_, *M*_*a*_ and *M*_*b*_ are the methods applied for a and b, *U*_*x*_ and *U*_*y*_ are two sets of users, developers and business experts. The hypothesis provides a mathematical framework for a robust implementation of different phases of CRISP-DM to ensure the update of different phases if the null hypotheses is invalidated.

Instead, in this paper considering the null hypothesis in Eq. 1, we discuss the interrelation of the Knowledge Discovery and Data Mining (KDD) framework for developing ML models and the transparency issues because a KDD model (Cios et al., 2012) which does not address various levels of details of business and data understanding for the development of ML models will be serving as an ad-hoc model, which can not be useful in fulfilling higher business objectives and decision making in the long term.

In this paper, we review the general CRISP-DM framework and discuss extensions thereof. A special focus of attention is placed on robustness-issues of ML and AI models for data from manufacturing and production within this framework, which is strongly related to model assessment. Furthermore, we emphasize the interplay between three parties, i.e., data experts, business experts and users, after deployment of a model. This human-centered aspect requires additional measures, e.g., for model transparency and model security frequently overlooked.

The paper is organized as follows. In the next section, we discuss the CRISP-DM framework in detail and suggested extensions. In order to simplify this discussion we will introduce a summarization of such a model we call Generalized Cross-Industry Standard Process for Data Science (GCRISP-DS). Next, we discuss the meaning of a human-centered data science and its role as a safety system. Finally, we discuss the problem of model assessment and data-related robustness issues of machine learning models which are critical for successful project implementations. This paper finishes with a discussion and conclusions.

## Cross-Industry Standard Process for Data Mining and Extensions

The *Cross-Industry Standard Process for Data Mining* (CRISP-DM) framework was introduced in 1996. Its goal is to provide a systematic and general approach for applying data mining concepts to analyze industrial operations and gain in-depth insights into business processes (Shearer, 2000). It is a widely accepted framework for industrial data mining and data analytics for data-driven knowledge discovery. The CRISP-DM framework is an endeavor to provide a general framework that is independent of any given industry and of applications that execute data mining methods that look at different stages of a business (Shearer, 2000). Briefly, CRISP-DM divides the data mining-related knowledge discovery process into six phases: 1) business understanding, 2) data understanding, 3) data preparation, 4) modeling, 5) evaluation and 6) deployment.

The different phases of CRISP-DM and the underlying associated tasks are shown in Table 1. The added third column provides a brief description of the literature, which explicitly reviews the challenges of the corresponding component related to industrial production and Big Data.

**TABLE 1:** Different components of CRISP-DM model and the associated tasks.

CRISP Components	Tasks	Literature and Description
Business understanidng	–Define business objectives – Risk Assessment analysis –Cost and benefit analysis –Technical requirement analysis – Define data analysis objectives and project planning	In Sharma and Osei-Bryson (2009) a framework for implementing various business understanding tasks is presented and highlights dependencies between them. In Sharma and Osei-Bryson (2008); Rao et al. (2012) an organizational-ontology for business understanding is presented. In Nino et al. (2015) various aspects of business understanding and challenges related to big data are discussed
Data understanding and preparation	– Data extraction – Data description – Data quality estimation – Data selection for modeling Data cleaning and feature extraction – Data exploration	In Duch et al. (2004) rule-based data extraction and understanding is discussed. Uddin et al. (2014) discuss various characteristics of big data for its efficient applications. Karkouch et al. (2016); Qin et al. (2016) discuss data properties, life-cycle of data from internet of things (IoT) for maintaining data quality from IoT. Cichy and Rass (2019) reviews various comparisons that provide data quality frameworks from different areas, including industrial production. Hazen et al. (2014); Ardagna et al. (2018) discuss methods for data quality management, monitoring, and assessments. Steed et al. (2017); Zhou et al. (2019); Andrienko et al. (2020)) discuss visualization methods and challenges of manufacturing and big data. Stanula et al. (2018)) discuss guidelines for data selection for understanding business and data in manufacturing
Modeling and evaluation	– Model assumption and selection techniques for modeling, parameter selection – Feature engineering – Model testing, result visualization and analysis – Model evaluation and description – Other data and modeling issues affecting model performance	In Diez-Olivan et al. (2019), Vogl et al. (2019), Bertsimas and Kallus (2020) reviews of various models, models building and evaluation for descriptive, diagnostic, predictive, and prescriptive analysis in industrial production and manufacturing are presented
Deployment	– Model utility assessment – Model monitoring, maintenance and updates – Users response evaluation – Model evaluation for data understanding and business understanding	Issues of model deployment related to human-lefted data science and model safety are discussed in *HUMAN-CENTERED Data Science and Model Safety*

**FIGURE 1:**
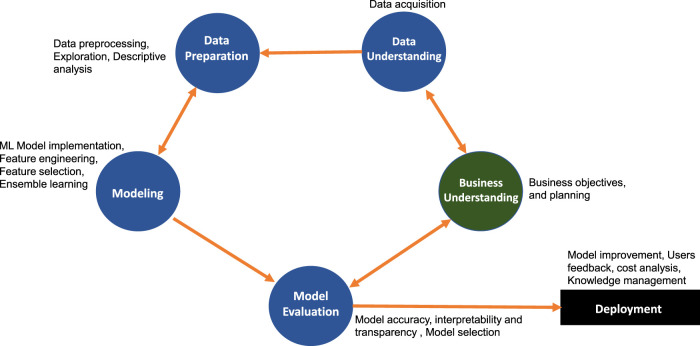
The CRISP-DM framework describes a cyclic process of a data analysis project.

In Figure 1, we show a schematic overview of the standard CRISP-DM framework. The edges are the interactions on between different phases of CRISP-DM. For example, the modeling phase depends on data preparation and vice versa. When the data preparation phase is complete, the next step is the modeling phase for which different candidate models with different assumptions about the data are proposed. To meet the data requirements for model building, data experts might step back to the data preparation phase and apply various data preprocessing methods. Similarly, in another example, the data acquisition and data preprocessing steps are done by data experts so that business goals can be achieved with the available data. Business experts and data experts can agree to obtain the data through a specific experimental design or by acquiring new observational data.

Importantly, business goals may need to be readjusted based on an understanding acquired during the data preparation and data understanding phases. The readjustment of business goals may imply the required time, cost, data quality and the usefulness of the business plan. Once the data experts and business experts agree on data issues, the data analysis process moves forward to model development and evaluation. Similarly, also these phases require the cooperation between different groups. Overall, the standard CRISP-DM describes a cyclic process, which has recently been highlighted as the emergent feature of data science (Emmert-Streib et al., 2016). In contrast, ML or statistics focus traditionally on a single method for the analysis of data. Thus, the CRISP-DM framework follows a general data science approach.

**FIGURE 2:**
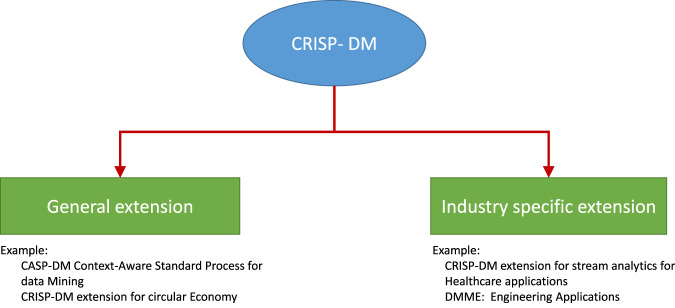
Classification of the CRISP-DM framework into two categories: general extensions and industry-specific extensions.

In Figure 2, we highlight two types of CRISP-DM models: general extensions (aka concept-based extensions) and industry- (or application) specific extensions. For the circular economy, KDD and CRISP-DM are the concept-based evolution of the CRISP-DM model. However, *Data Mining Methodology for Engineering* (DMME) and CRISP-DM for stream analytics are application-specific customizations of the basic model. Given the new industrial trends, concept-based evolution has become a generalized extension of CRISP-DM. For example, the circular economy is a growing trend in the industry for lifelong services, product upgrades, and product recycling for sustainable and environmentally conscious manufacturing (Walter, 2016). Hence, all business sectors should gradually adopt the circular economy concept. Therefore, a knowledge discovery model must adopt concept-related extensions of the CRISP-DM framework.

It is important to note that the standard CRISP-DM framework developed in 1990s did not envisage the big data evolution and futuristic applications of data-driven models for automated decision making where the data dimensions, speed and other big data characteristics play key roles for determining the efficiency of data mining and ML models. Therefore the standard model suffers from a number of limitations and various required extensions of CRISP-DM and data mining algorithms, models, and techniques were developed over the years to achieve robustness in advanced automated decision-making processes (Liu et al., 2018).

A recent example of such an expansion is the *Data Mining Methodology for Engineering* applications (DMME) (Huber et al., 2019) model, which is an extension of the CRISP-DM model for engineering applications. This model adds three further phases, namely, technical understanding, technical realization (which sits between business understanding and data understanding), and technical implementation (which sits between the evaluation and deployment phases). The DMME model also draws new interactions between different phases, e.g., between evaluation and technical understanding, between data understanding and technical understanding, and between technical understanding and technical realization. Furthermore, this model also emphasizes the cooperation between specialized goals for the refinement of the framework.

Similarly, in light of big data, technical advancements, broader business objectives, and advanced data science models, Grady (Leslie, 1978) discussed the need for a new and improved framework. The author coined the term *Knowledge Discovery in Data Science* (KDDS) and discussed different aspects of KDDS, such as KDDS phases, KDDS process models, and KDDS activities. Grady emphasized that this approach establishes a new and improved framework, which he calls the Cross-Industry Standard Process model for Data Science (CRISP-DS). Table 2 lists further extensions of the CRISP-DM model. These models show that the basic CRISP-DM framework cannot satisfy the variety of data mining requirements from different sectors.

**TABLE 2:** List of extensions of the CRISP-DM process model framework based on industry-specific requirements (application-specific requirements) due to changing business trends.

Model	Description	Application
DMME (Huber et al., 2019)	Adds two new phases between business understanding and data understanding and one phase between model evaluation and deployment	Engineering applications
KDDS with big data (Grady, 2016)	Adding various new activities, especially to handle big data	A proposed framework as a need for the current scenario in big data and data science applications
CRISP-DM extension for stream analytics (Kalgotra and Sharda, 2016)	Data preparation and data modeling stage to be redefined for multidimensional and time-variant data, where the IoT system sends multiple signals over time	Healthcare application
CRISP-DM extension in context of circular economy (Kristoffersen et al., 2019)	Adds a data validation phase and new interactions between different phases	Aligning business analytics with the circular economy goals
Context-aware standard process for data mining (CASP-DM) (Martínez-Plumed et al., 2017)	The deployment context of the model can differ from the training context. Therefore, for context-aware ML models and model evaluation, new activities and tasks are added at different phases of CRISP-DM.	Robust and systematic reuse of data transformation and ML models if the context changes
APREP-DM (Nagashima and Kato, 2019)	Extended framework for handling outliers, missing data, and data preprocessing at the data-understanding phase	General extension for automated preprocessing of sensor data
QM-CRISP-DM (Schäfer et al., 2018)	CRISP-DM extension for the purpose of quality management considering DMIAC cycle	Adding quality management tools in each phase of CRISP-DM framework validated in the real-world electronic production process
ASUM-DM (Haffar, 2015)	IBM-SPSS initiative for the practical implementation of the CRISP-DM framework, which combines traditional and agile principals. It implements existing CRSIP-DM phases and adds additional operational, deployment, and project-management phases	General framework that allows comprehensive, scalable, and product-specific implementation
A variability-aware design approach for CRISP-DM (Vale Tavares et al., 2018)	Extends the structural framework to capture the variability of data modeling and analysis phase as feature models for more flexible implementation of data process models	General framework which considers model and data variability for the improved automation of data analysis

**FIGURE 3:**
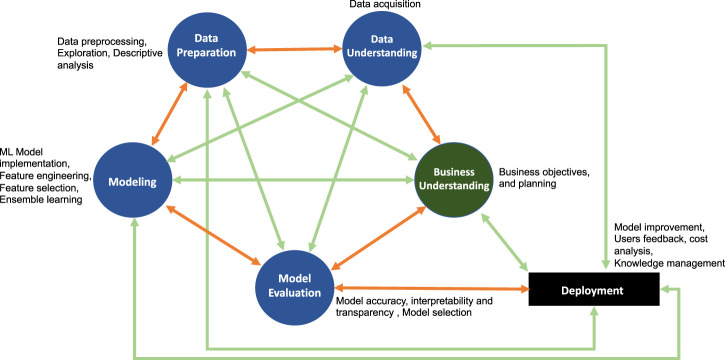
Generalized Cross-Industry Standard Process for Data Science (GCRISP-DS). This framework is designed to allow dynamic interactions between different phases to adequately address data- and model-related issues for achieving robustness.

In general, the extensions of CRISP-DM shown in the Table 2 extend the standard framework to fulfill specific requirements. Various expansions add new phases or new attributes or activities on the existing phases. Subsequently, this added new interactions between CRISP-DM phases as well as new proposed phases. Summarizing all these extensions into one coherent framework one obtains a fully connected CRISP network as a generalized abstract framework of the various steps constituting a systematic analysis and knowledge discovery. This extension is shown in Figure 3 and we call this the *Generalized Cross-Industry Standard Process for Data Science* (GCRISP-DS).

In Figure 3, the orange edges are from the traditional CRISP-DM whereas the green ones are newly added layers of complexity and interactions. The fully connected GCRISP-DS raises new questions about how these edges are useful and what new information they contributes? In our understanding the six basic phases of CRISP-DM must be interacting actively which means that the business experts, data experts and data scientists actively collaborate to evaluate business problems and the data-driven solutions. Therefore, the active collaboration would ensure if a new phase or interaction is required between existing phases or a new attribute to be added in different phases. Also, it should ensure how effectively the interactions should stay between phases. Thus, with active collaborations one can draw a new specific subclass of a GCRISP-DS. The new specific model (subclass or extended subclass of fully connected GCRISP-DS) would be an adaptation to the companies’ business environment and solutions. We want to emphasize three main points for active collaboration between all possible interactions between phases:

Evaluate requirements of extensions of existing phases, activities and attributes depending on technical and other business specific needs for adopting industrial application-specific requirements or general changes in the economy or manufacturing.
Ensure the models’ utility and robustness with respect to external changes in the business processes, data quality and business understanding.
Ensure the model transparency, explainability and reusability for newer business understanding by considering users’ response to the deployed model.

## Human-Centered Data Science and Model Safety

The new wave of AI and ML solutions aims to replace human-related tasks and decision making processes by automated methods. This raises concerns regarding responsible and ethical ML and AI models that do not underestimate human interests and do not incorporate social and cultural bias. ML systems have, until now, primarily focused on applying cost-effective automations that are useful for business organizations, but such automations are so far not part of complex decision making processes. However, in the future, autonomous systems could take over for many crucial decision making processes. One example of such an automation are self-driving cars. Many large car manufacturing companies have announced the launch of self-driving cars, but the acceptability and use of such vehicles is a major challenge (Urmson and Whittaker, 2008; Howard and Dai, 2014). (Kaur and Rampersad, 2018) quantitatively analyzed the acceptance of technology that addresses human concerns. Their study analyzed the public response to this issues, which include security, reliability, performance expectancy, trust or safety, and adoption scenarios.

Similarly, such cases can be discussed for industrial production and manufacturing problems when a self-automated system or a self-functioning machine based on AI or ML decides various production and manufacturing tasks. In these cases, how a system would function in complex scenarios would be a question, both ethical and technical. This type of autonomy can affect the complex production process of PPCSs estimation, which might, in adversarial cases, influence the robustness and stability of PPCSs, predictive maintenance, and other production processes (Oliveira et al., 2019). Another future question one can ask is, what should be done with an automated system after it is deployed? Should it be left with no human intervention, should it always supersede human understanding, and how can humans and AI systems cooperate effectively?

In industrial production cases, human-centered data science issues can be seen from the following perspectives:

• What is the role of human-centered ML and data science processes in decision making related to work goals, business goals and societal goals?
• Can production processes be completely automated with no involvement of humans by AI agents with human-like intelligence?
• The emergence of complexity when a series of tasks is automated and integrated: Can an AI or ML system exhibit the higher level of intelligence required for independent and integrated decision making?

When industrial production processes are completed by a combination of humans and ML agents: How can humans and ML agents interact effectively for integrated analysis and decision making?

The first point mentioned here is still in its infancy and awaits the future of integrating complex data mining and ML processes. The future design of data science modeling of complex data and business goals is beyond the scope of this paper. However, a fundamental question that remains relevant is how can large-scale automation led by AI and ML models impact humans? At its core, this question probes how AI can efficiently serve, empower, and facilitate human understanding and decision making tasks and provide deeper insight into the solutions to these complex problems. This question also leads to the second and third points; namely, should we leave the decision making entirely in the hands of AI-ML models? In other words, can a machine decide by itself what is right or wrong for humans? Complete automation would lead to the emergence of many complex aspects of the production process, and dealing with such complexity would be the future challenge of research into production and manufacturing. In such complex cases, the role of humans should be to interact and collaborate with the automated systems. They should use intelligent models for decision making and have the wisdom to override machine decisions if required for ensuring safety.

**FIGURE 4:**
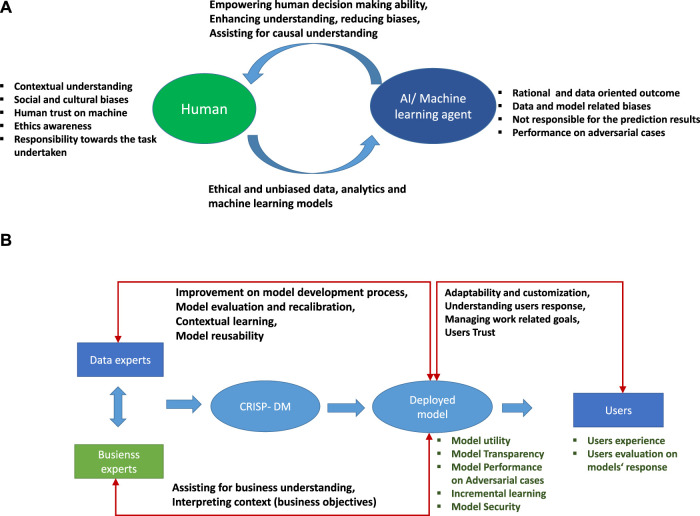
**(A)** Schematic view of interactions between humans and AI and ML agents. **(B)** Schematic view of post-deployment of ML model not left in isolation but continuously updating and addressing human concerns by allowing human interactions to ensure a human-centered data science.

Importantly, there is a difference between a human perspective and statistical learning perspective because both have different characteristics and capabilities. A ML model makes decisions based on a narrow data-related view with no or limited contextual understanding. The AI-ML models cannot be held responsible for their decisions. However, they can produce results rationally and logically based on the data used to train them. Interactions between humans and machines are possible in two ways: The first way involves the model developer and ML models, where the training of models are the responsibility of data experts and business experts. They should train models with a diverse range of data with no bias and no breach of data ethics. The second way is between model users and ML models. The results predicted by models empower and assist humans to make decisions regarding simple to complex tasks. Figure 4A shows a schematic view of human-machine interactions. In this figure, we highlight various characteristics of human and ML models and how effective interactions between humans and ML models complement and empower human decision making and further improves the capacity of models to make fair analyses.

Further extending the human-machine interaction concept, we now discuss the industrial-production framework shown in Figure 4B. This highlights a CRISP-DM process in the deployment phase of the model. The deployed model should have proven its utility and allow transparency so that the user can understand the models’ prediction and trust the results. Importantly, the deployed model interacts with data experts, business experts, and actual users. In an industrial production process, the users are technical operators or machine handlers. These interactions have different meanings for different users. Data experts interact with the model to improve the prediction accuracy and model performance. They provide contextual meaning to the results predicted by the model and train it further for contextual learning. The data experts also explore the reusability of a model for other cases (Pan and Yang, 2010). Business experts should test these results against their understanding of larger business goals and should explain the business context based on model results. Furthermore, the results should allow the model to be reused and adaptable to changes in production and manufacturing scenarios.

In production and manufacturing, data-driven knowledge processes must adopt human-centered concerns in data analytics and ML models; the human-machine interaction must not fizzle out after the deployment of the model (Amershi et al., 2014). The model should remain interactive with its users and be allowed to evolve and update itself. Other characteristics of the deployed models are its transparency and performance in adversarial cases. Model transparency should enable users to access various model features and evaluate the predicted results. Additionally, the human-machine interaction must be aware of the surrounding environment so that the model can adapt to environmental changes, as required for intelligent learning of ML models. Continuous interactions between humans and machines improve the generality of the model by incremental learning and updating of the model so that it gives robust and stable responses and can be adaptive to changes.

## Model Assessment

For the remainder of this paper, we focus on two interrelated aspects of GCRISP-DS or CRISP-DM, namely, model assessment and model robustness. While all phases and interactions are important the effect of improper models or lack of robustness may lead to the most devastating problems of the analysis pipeline. For this reason, we discuss model assessment and model robustness issues in detail to emphasize their importance. With respect to Figures 1 and 3 these correspond to the internal structure of the corresponding phase nodes.

Model building and evaluation in CRISP-DM are two phases in which data are 1) searched for patterns and 2) analyzed. These models are divided into four classes: descriptive, diagnostic, predictive, and prescriptive (Bertsimas and Kallus, 2020). The primary goal of these models is pattern recognition, machine-health management, condition-based maintenance, predictive maintenance, production scheduling, life-cycle optimization, and supply-chain management prediction. Alberto et al. (Diez-Olivan et al., 2019) provide a comprehensive review of various methods applied in manufacturing and production with representative studies of descriptive, predictive, and prescriptive models of various industrial sectors, including manufacturing. Vogl et al. (2019) review current practices for diagnostic and prognostic analysis in manufacturing and highlight the challenges faced by the prognostic health management system in the current scenario of automated maintenance.

**FIGURE 5:**
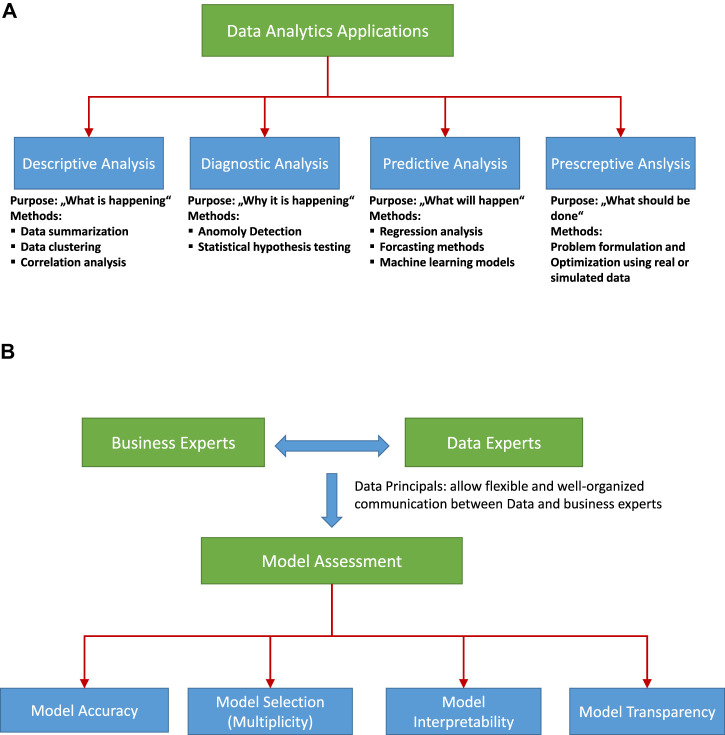
**(A)** Components of data analytics problems. **(B)** Model assessment.

Figure 5A shows a schematic view of the division of data analysis problems. The divisions are useful to understand the nature of the problem and the methods that are applicable to the problem.

**FIGURE 6:**
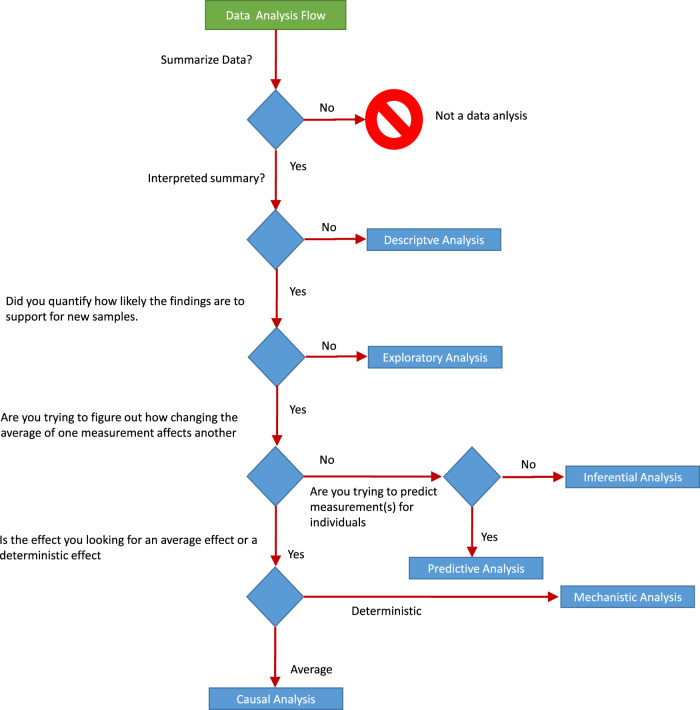
Schematic diagram showing data analytics process. Connections are shown between the individual analysis steps that constitute the whole project Lee et al., 2019.

Leek and Peng (2015) emphasizes that, even if the statistical results are correct, data analysis can be wrong because the wrong questions are asked. One must be aware of such a situation in an industrial framework and should select the right approach for the analysis. He further divides data analysis into six categories (see Figure 6). This chart is useful for the basic understanding of data analysis for business experts and data experts, which helps prevent knowledge discovery from diverging from the real objective.

The business understanding and the later data modeling approaches for solving the type of problem shown in Figure 5A requires a systematic interaction between data experts and business experts. Such communication is possible only when the data experts and business experts are aware of each other and agree with each other. Therefore, they need to address the data- and model-related communication in the context of business understanding. Hicks and Peng, (2019) discuss the vocabulary required to convey the accurate meaning of the data analysis. Additionally, They describe the six principles of data analysis: Data matching, Exhaustive, Skeptical, Second order, Transparent, and Reproducible. These six principles are applicable to an industrial framework to communicate the results of the data analysis. A clear understanding of these principles helps business experts and data experts develop robust models for data-driven knowledge discovery. Data- and model-related issues are not restricted only to understanding business and data; the most crucial part is model assessment, which requires accurate nonambiguous model selection, interpretability, and transparency. Model assessment depends on various issues, namely, model accuracy, model multiplicity, model interpretability, and model transparency (see schematic diagram in Figure 5B), and various strategies can be applied to obtain the best model assessment. Model assessment requires an agreement between data and business experts, so all four components of model assessment shown in Figure 5B should be addressed properly. The first part is the model accuracy; Raschka, (1811) reviews model evaluation, selection, and ML model selection for various ML methods, and Palacio-Niño and Berzal (Berzal, 2019) review the evaluation of unsupervised learning methods. Akaike information criterion (AIC), Bayesian information criterion (BIC), cross-validation error, and probabilistic model selection are some of the approaches used to select less complex models. In (Emmert-Streib and Dehmer, 2019b) key statistical characteristics for systematically evaluating regression models are discussed with respect to model-complexity for selecting the best model. Model interpretation is another crucial issue in data-driven knowledge discovery with ML models. In general, monotonically increasing functions (linear and nonlinear), such as additive models, sparse linear models, and decision trees, are regarded as interpretable models, whereas nonlinear and nonmonotonic functions have higher complexity and thus a lower interpretability (Ribeiro et al., 2016a; Hall and Gill, 2018). (Doshi-Velez and Kim, 2017) provides a theoretical framework for the interpretability of models by defining three classes for evaluating interpretability: The first class is application-grounded evaluation, where the domain experts evaluate the model with real tasks. Next, human-grounded evaluation allows a model to be tested with simplified tasks without a specific end-goal. The third class is functionally grounded evaluation, which uses a predefined definition of interpretability of a model as proxy and optimizes the candidate models. In an industrial setup, most of the model evaluations involve functionally grounded evaluation. Application- and human-grounded evaluation requires more resources and could be expensive and time consuming. Functionally grounded evaluation can be useful for learning if the selection of proxy models is based on factors that are relevant to real-world applications.

Model agnostic interpretability is another approach for interpreting ML models. In this approach, a model is treated as a black box, and the model is analyzed by entering perturbed input data and analyzing the respective output (Ribeiro et al., 2016a). The model agnostic approach allows the application of a wide range of black-box ML models as interpretable for predictive analysis. Local interpretable model-agnostic explanations (Ribeiro et al., 2016b) explain a model locally by constructing a local binary classifier of a regularized linear model by using simulated or perturbed input data to explain an instance predicted by the black-box machine learning model.

Another issue with model assessment is the multiplicity of models; such cases are known as the “Rashomon” effect (Breiman, 2001), whereby multiple predictive models make predictions with competing accuracy of the same target variable. In such cases, one should not come to a conclusion about the validity of a prediction in terms of its explanatory variables from one or a few models until the hypothesis of model multiplicity is invalidated. In industrial scenarios, the Rashomon effect can occur when we have multiple models with competing accuracy; one can then choose a narrative to interpret the results based on the selected model. Such interpretations can differ for different models and would impact the business objective or business understanding. Fisher et al. (2018) proposes the model class reliance, which is a measure of a set of variables of a model that show high predictive accuracy. Marx et al. (Charles et al., 2019) propose the ambiguity and discrepancy measures to evaluate the multiplicity problem in classification models. In this approach, models are selected from a set of “good” models, which maximizes the discrepancy compared to a proposed baseline model. To select a model with simplicity and accuracy, Semenova and Rudin (2019) propose the *Rashomon ratio* measure, which is a ratio of competing models in hypothesis space.

In a business scenario, when manufacturing becomes increasingly complex and integrated, data-driven models are useful to make informed decisions and automate functioning, which allows for efficient and cost-effective production. Even for a model with high accuracy, user trust is always a crucial consideration for the deployment of a model. In such cases, transparency is always a matter of concern and should be addressed with simple explanations. Trust issues arise because of data-source uncertainty, the performance of deployed models, and user belief in the model output. This uncertainty can seep into the whole process of data mining, from data preprocessing to model building, and thus impacts the entire business-intelligence process. Sacha et al. (2015) provide a framework to address human-machine trust issues that are caused by uncertainties propagated in data processing, model building, and visual analytics. The proposed framework to deal with the uncertainty is divided into two parts: The first part is the system-related uncertainty and emphasizes handling machine uncertainty by quantifying uncertainty in data processing and model building, aggregation of total uncertainty at different phases of data analysis, and interactive exploration of uncertainty through visual and other means. The second part involves human factors that emphasize transparency, which allows system functions to access experts and users for review and to explore uncertainty, thus building an awareness of the emerging uncertainty—and additionally tracking human behavior, which is also useful to estimate human bias and how it impacts human-machine interactions.

Data transparency and model transparency should ensure that the entire process is explainable to business experts and users. It should also be secure from external attacks, which allows models to be deployed for making business decisions, for user control, and for social acceptance of intelligent systems Weller, 2017; Springer, 2019. Emmert-Streib et al. (2020b) featured that the AI models help analyze data but not establish a scientific-theory of the system that provides the data. Therefore, methods like deep learning should not be applied as a first choice for the tasks where requirements for explainable models are essential. However, these models should be utilized for comparison with the other nearest explainable models for evaluation. Weller (2017) discusses eight types of transparency rules for developers, business experts, and users to help them understand the existing system functioning. Transparency allows a large and complex system to be robust and resilient and self-correcting, and ultimately ensures steady and flexible models.

## Robustness Issues of ML and AI Models

In the following, we discuss data-related issues for robust data analytics because the shortcomings of data reflect on model evaluation and on the deployment phase in terms of underperformance and biased predictions of real-world problems.

### Experimental Design and Sample Size

In data-driven process optimization, the data may be observational data or experimental data. Experimental data are used to test the underlying hypothesis to build an understanding and to optimize the response variable by controlling or modifying various combinations of input parameters. In manufacturing, the experimental designs mostly focus on optimizing various parameters for quality control. The experimental design is a key criterion for determining whether a model employs all the right answers to be understood in ML-based analysis and manufacturing optimization. The production process is controlled by setting various process variables, which, during production, are monitored for changes. In an experimental design, we monitor certain process variables by controlling other variables to understand how they affect product quality. The one-factor approach is common and involves repeating experiments with the factors changed between each experiment until the effect of all factors is recorded. However, this one-factor approach is time consuming and expensive. For a robust experimental design, Taguchi proposed a methodology that uses orthogonal arrays, signal-to-noise ratios, and the appropriate combination of factors to study the entire parameter space through a limited number of experiments (Taguchi and Konishi, 1987; Taguchi et al., 1989; Unal and Dean, 1991; Chang and Faison, 2001). An optimal experimental design is a useful strategy that stands between business and data understanding. This approach also benefits model robustness in the evaluation phase in the CRISP-DM framework. The active interaction between business understanding and data understanding serves to solve the optimal sample-size issue and prevent an under-powered analysis.

### Model Complexity

The complexity of predictive models is sensitive to the prediction of testing data. Any complex model can lead to overfitting, whereas simpler models lead to prediction bias. This property of predictive models is known as “bias-variance trade-off.” Complexity in the model could lead to robustness issues, such as large testing error or the wrong interpretation of model parameters. In some cases, if future data contain a lower signal-to-noise ratio, large prediction error may occur. This problem can arise because of a large number of correlated features or through feature engineering, where we create a large number of redundant features with high correlation or when we try to fit a high-degree polynomial model in our data set.

Vapnik Chervonenk (VC) dimension (Vapnik and Chervonenkis, 1971) is a quantitative measure for measuring the complexity of models and is used to estimate the testing error in terms of model complexity. A model is selected based on the best performance with testing data and, as the complexity increases, the optimized training error decreases and the expected testing error first increases, then decreases, and then increases again. The training error and VC are used to calculate the upper bound of the testing error. This method is called “structural risk minimization” and is a model that minimizes the upper bounds for the risk. Regularization (Tibshirani, 1996; Chang and Faison, 2001; Yuan and Lin, 2006; Emmert-Streib and Dehmer, 2019c) and feature-selection methods (both wrapper- and filter-based) (Maldonado and Weber, 2009; Ko et al., 2017) are the useful strategies of variable selection to keep model complexity in check by minimizing bias and variance. Hoffmann et al. (2015) propose a sparse partial robust *M* regression method, which is a useful approach for regression models to remove variables that are important due to the outlier behavior in the data. The model complexity affects the modeling and model evaluation, such as model description, robustness, and parameter settings in the CRISP-DM framework.

### Class Imbalance

In production and manufacturing data, class imbalance is a common phenomenon. One such example is vibration data from cold testing of engines: all manufactured engines go through cold testing, and the technical expert tries to identify different errors in the engines by using strict threshold criteria. They label any errors found with a particular error label based on their technical knowledge. The proportion of engines with errors and the number of good engines are very low. To implement a multiclass classification model using previous data, which automatically identifies the error class of an engine, developing such models with high accuracy is difficult. Skewed sample distributions are a common problem in industrial production-related quality control, and imbalanced data lead to models where predictions are inclined toward the major class. In such cases, the major class is “good engines.” If a model classifies a faulty engine as a good engine (high false positives), then it would severely affect the reputation of quality control. In such cases, false positives could severely affect product reputation. Undersampling of major classes and oversampling of minor classes form the basis of the solution commonly proposed for the problem. References (Tajik et al., 2015; Zhang et al., 2015; Duan et al., 2016) discuss fault detection models with an imbalanced training dataset in the industrial and manufacturing sector. Resampling methods (oversampling, undersampling, and hybrid sampling) (Chawla et al., 2002; Han et al., 2005; Cateni et al., 2014; Sun et al., 2015; Nekooeimehr et al., 2016), feature selection and extraction, cost-sensitive learning, and ensemble methods (Krawczyk, 2016; Guo et al., 2017) are other approaches to deal with the class-imbalance problem. The use of evaluation measures considering the presence of undersampled classes is also recommended to evaluate models, such as the probabilistic thresholding model (Su and Hsiao, 2007), adjusted *F* measure (Maratea et al., 2014), Matthews correlation coefficient (Matthews, 1975), and AUC/ROC. The imbalance is critical to model building and evaluation problems in the CRISP-DM framework.

### Data Dimensionality

In ML and data mining, high-dimensional data contain a large number of features (dimensions), so the requirement of optimal samples increases exponentially. Therefore, a high-dimensional dataset is sparse (*p*≫*n*). In modern manufacturing, the complexity of the manufacturing processes is continuously increasing, which also increases the dimensionality of process variables that need to be monitored. Wuest et al. (2014) discuss manufacturing efficiency and product quality by considering the complexities and high dimensionality of manufacturing programs. High-dimensional data are a challenge for model robustness, outlier detection (Arthur et al., 2012), time, and algorithmic complexity in ML. These higher-dimensional data require changes in existing methods or the development of new methods for such sparse data. One approach is to use a dimensionality-reduction strategy such as a principal component analysis (Subasi and Ismail Gursoy, 2010) and use the principal components for ML methods. Such a strategy also requires a careful examination because of information loss when data are projected onto lower dimensions. Similarly, another strategy is feature selection, where the most important feature variables are found and we remove the redundant features from the model. Regularization (Tibshirani, 1996), tree-based models (Hsu, 2004; Bertsimas and Dunn, 2017; Guolin et al., 2017), mutual information (Jorge and Pablo, 2014; Bennasar et al., 2015), and clustering are other approaches for feature selection.

### Data Heteroscedasticity

In regression problems, the underlying assumption *ŷ* for the error term in the response data is that it has constant variance (homoscedasticity). Although such assumptions are useful to simplify models, real-world data do not follow the assumption of the constant variance error term and thus violate the assumption of homoscedasticity. Therefore, real data are heteroscedastic. Predictions based on a simple linear model of such data would still be consistent but can lead to practical implications that produce unreliable estimates of the standard error of coefficient parameters, thus leading to bias in test statistics and confidence intervals. Heteroscedasticity can be caused directly or indirectly by effects such as changes in the scale of observed data, structural shifts in the data and outliers, or the omission of explanatory variables. Heteroscedasticity can be parametric, which means that it can be described as a function of explanatory variables (White, 1980), or unspecified, meaning that it cannot be described by explanatory variables. Examples in manufacturing are related to predictive maintenance (i.e., predicting the remaining useful life of a machine based on vibration data), quality control, and optimization (Tamminen et al., 2013; Lee et al., 2019). Heteroscedasticity in regression models is identified by analyzing residual errors. Residual plots and several statistical tests may be used to test for heteroscedasticity in data (Leslie, 1978; Breusch and Pagan, 1979; White, 1980; Markowski and Markowski, 1990). Other studies and methods to model the variance of response include weighted least squares (White, 1980), the heteroscedastic kernel (Cawley et al., 2004), heteroscedastic Gaussian process regression (Kersting et al., 2007; Kotanchek et al., 2010), heteroscedastic Bayesian additive regression trees (BART) (Pratola et al., 2017), and heteroscedastic support vector machine regression (Hu et al., 2016).

### Incomplete and Missing Data

Data quality is an essential part of data-driven process optimization, process quality improvement, and smart decision making in production planning and control. In the manufacturing process, data come from various sources and is heterogeneous in terms of variety and dimensionality. Data quality is one of the important issues for the implementation of robust ML methods in process optimization. Data quality may suffer for various reasons such as missing values, outliers, incomplete data, inconsistent data, noisy data, and unaccounted data (Köksal et al., 2011). The typical way to deal with missing data is to delete them or replace them with the average value or most frequent value and apply multiple imputations (Fritz, 2005) and expectation maximization (Allen and Tibshirani, 2010). Another method is to consider the probability distribution of missing values in statistical modeling (Rubin 1976; Little and Rubin, 2019). These considerations are of three types: 1) missing completely at random, 2) missing at random, and 3) missing not at random. Joint modeling and fully conditional specifications are two approaches to multivariate data imputation (Van Buuren et al., 2006; van Buuren, 2007). Recent studies to impute missing data in production and manufacturing and optimize production quality considered classification and regression trees to impute manufacturing data (White et al., 2018), modified fully conditional specifications (Guan et al., 2017), and multiple prediction models of a missing data set to estimate product quality (Kang et al., 2018). Loukopoulos et al. (2018) studied the application of different imputation methods to industrial centrifugal compressor data and compared several methods. Their results suggest multiple imputations with self-organizing maps and *k* nearest neighbors as the best approach to follow. Incomplete and missing data have repercussions in business and data understanding, exploratory analysis, variable selection, model building, and evaluation. A careful strategy involving imputation or deletion should be adopted for missing data.

### External Effects

External effects are important factors in data-driven process optimization. In a big data framework, external effects might be unnoticed, incorrectly recorded, or not studied in detail (i.e., unknown). External effects can involve environmental factors (e.g., temperature, humidity), human resources, machine health, running time, material quality variation, and other factors that are dealt with subjectively but significantly affect production and data quality. For example, to create a ML model to predict product quality, the data (process parameters) are recorded by sensors from several machines. In the injection molding process, the process parameters cannot estimate product quality alone because product quality is significantly affected by temperature, humidity, machine health, and material type, and these external factors must be considered to optimize quality. Similarly, in the sintering process (Strasser et al., 2019), where product quality is predicted in terms of its shape accuracy, the data from various experiments show a considerable variation in different process parameters from unknown and unaccounted sources. In such cases, the prediction can be biased or erroneous with high variance. A careful examination involving exploratory analysis is required to detect such effects. Gao et al. (2017) studied a hierarchical analytic process to produce a comprehensive quality evaluation system of large piston compressors that considers external and intrinsic effects.

### Concept Drift

Changes in data over time due to various factors such as external effects, environmental changes, technological advances, and various other reasons emerge from unknown real-world phenomena. ML models, in general, are trained with static data and thus do not consider the dynamic nature of the data, such as changes over time in the underlying distribution. In such cases, the performance of these models deteriorates over time. Robust ML modeling requires identifying changes in data over time, separating noise from concept drift, adapting changes, and updating the model. Webb et al. (2016) provide a hierarchical classification of concept drift that includes five main categories: drift subject, drift frequency, drift transition, drift recurrence, and drift magnitude. They make the case that, rather than defining concept drift qualitatively, a quantitative description of concept drift is required to understand problems in order to detect and address time-dependent distributions. These quantitative measures are cycle duration, drift rate, magnitude, frequency, duration, and path length and are required for a detailed understanding of concept drift. Such an understanding allows modeling by new ML methods that are robust against various types of drifts because they can update themselves by active online learning and thereby detect drifts. Lu et al. (2018) provide a detailed review of current research, methods, and techniques to deal with problems related to concept drift. One key point in that paper is that most of the methods identify “when” a drift happens in the data but cannot answer “where” or “how” it happened. They further emphasize that research about concept drift should consider the following themes:

1) Identifying drift severity and regions to better adapt to concept drift;
2) Techniques for unsupervised and semisupervised drift detection and adaptation;
3) A framework for selecting real-world data to evaluate ML methods that handle concept drift;
4) Integrating ML methods with concept-drift methodologies (Gama et al., 2013) discuss the various evaluation and assessment practices for concept-drift methods and strategies to rapidly and efficiently detect concept drift).

Cheng et al. (Lin et al., 2019) present the ensemble learning algorithm for condition-based monitoring with concept drift and imbalance data for offline classifiers. Yang et al. (2019) discuss a novel online sequential extreme learning model to detect various types of drifts in data. Finally, Wang and Abraham (2015) proposed a concept-drift detection framework known as “linear four rates” that is applicable for batch and streaming data and also deals effectively with imbalanced datasets.

In the manufacturing environment, concept drift is an expected phenomenon. When discussing with technical experts, data drift should be understood and explained clearly by experts for data and business understanding to ensure that future models are flexible and adaptable to changes due to data drift.

### Data Labeling

Data annotations (i.e., labels) as output allow ML methods to learn functions from the data. Specifically, input data are modeled through class labels by using supervised ML models for classification problems. If the labeling is noisy or not appropriately assigned, the predictive performance of the classification model is severely degraded. Therefore, for supervised ML models, data labeling or annotation is a nontrivial issue for data quality, model building, and evaluation. The labeling can either be done manually by crowd sourcing or by using semisupervised learning, transfer learning, active learning, or probabilistic program induction (Zhou et al., 2017). Sheng et al. (2008) discuss repeated-labeling techniques to label noisy data. In a typical manufacturing environment, labeling is done by operators or technical experts; in some cases, the same type of data is annotated by multiple operators who work in different shifts or at different sites. Sometimes, the operators do not follow a strict protocol and label intuitively based on their experience. In such cases, data quality can suffer immensely. Inadequate learning from data may occur if a large number of samples is not available for a specific problem and the data labeling is noisy.

Data labeling is a part of the data understanding phase and should follow a clear and well-defined framework based on the technical and statistical understanding of the manufacturing and production processes and their outcome. Many manufacturing and production tasks are managed by machine operators, technical experts, engineers, and domain experts using measures and experience to optimize the quality of the output. Because these experts are well aware of the existing situation, they should be allowed to label data to produce robust ML models and maintain data quality.

### Feature Engineering

The deep learning framework (Wang et al., 2018; Emmert-Streib et al., 2020a) provides a promising approach for automated smart manufacturing. These algorithms undertake automatic feature extraction and selection at higher levels in the networks. They are used for numerous important tasks in manufacturing, such as fault detection (Lu et al., 2017) or predicting machine health (Deutsch et al., 2017). Wang et al. (2018) comprehensively reviewed deep learning methods for smart manufacturing. Importantly, deep learning models may suffer from the same issues as discussed in previous sections. Data complexity, model and time complexity, model interpretation, and the requirement of large amounts of data means that they may not be suited for every industrial production problem. Therefore, the demand remains strong in industrial production for traditional ML algorithms using feature-selection methods.

In ML problems, a feature is an attribute used in supervised or unsupervised ML models as an explanatory or input variable. Features are constructed by applying mathematical functions to raw attributes (i.e., input data) based on domain knowledge. As a result, feature engineering and extraction lead to new characteristics of an original data set (i.e., it constructs a new feature space from the original data descriptors). Features can be created by methods that are linear, nonlinear, or use independent component analysis. The main objective of feature-extraction techniques is to remove redundant data and create interpretable models to improve prediction accuracy or to create generalized features that are, e.g., classifier independent. In general, feature learning enhances the efficiency of regression and classification models used for predictive maintenance and product quality classification. In the manufacturing industry, feature engineering and extraction is a common practice for the classification of engine quality, machine health, or similar problems using vibration data. For instance, fast Fourier transform or power spectral density statistical features are standard feature extraction methods used for vibration data (Caesarendra and Tjahjowidodo, 2017).

Sondhi (2009) discusses various feature-extraction methods based on decision trees, genetic programming, inductive logic programming, and annotation-based methods. Interestingly, genetic programming in combination with symbolic regression searches for a mathematical equation that best approximates a target variable. Given its simple and understandable structure, this approach has been useful for many industrial applications of modeling and feature extraction (Kohavi and George, 1997; Yang et al., 2015; Strasser et al., 2019). However, training for a large number of variables, model overfitting, and the time complexity of the model are problematic for this approach. Caesarendra and Tjahjowidodo (2017) discuss five feature categories for vibration data:

1. phase-space dissimilarity measurement;
2. complexity measurement;
3. time-frequency representation;
4. time-domain feature extraction;
5. frequency-domain feature extraction.

These feature engineering methods are useful to summarize various characteristics of vibration data and reduce the size of high-dimensional data. Mechanical vibration is a useful indicator of machine functioning. The features of vibration data are often used to train ML models to predict fault detection, do predictive maintenance, and for various other diagnostic analyses. He et al. (2019) discuss the statistical pattern analysis framework for fault detection and diagnosis for batch and process data. In this approach, instead of process variables, sample-wise and variable-wise statistical features that quantify process characteristics are extracted and used for process monitoring. This approach is useful for real-world dynamic, nonlinear, and non-Gaussian process data, which are transformed into multivariate Gaussian-distributed features related to process characteristics that are then used for multivariate statistical analysis. Ko et al. (Köksal et al., 2011) discuss various feature-extraction and -engineering techniques for anomaly detection or binary classification in different industries.

Recent results for deep learning networks and support vector machines demonstrate that feature selection can negatively affect the prediction performance for high-dimensional genomic data (Smolander et al., 2019). However, whether these results translate to data in other domains remains to be seen. Until then, feature selection remains a standard approach of data preprocessing.

In general, for the CRISP-DM framework, feature engineering is essential for real-world problems of model building. Real-world data features are not independent—they are correlated with each other, which hints of interactions between the underlying processes in manufacturing. Many of the higher-level effects related to the maintenance and product quality are combinations of lower-level effects of several processes and are reflected by the data. Therefore, approaches that use feature engineering, feature extraction, and feature selection are useful to build models to predict or test for higher-level effects.

## Discussion

In this paper, we reviewed some of the key issues affecting the performance, implementation and use of AI and ML models. Specifically, we discussed existing methods and future directions of model building and model evaluation in the area of smart manufacturing. In this context, GCRISP-DS is a general framework that provides a structured approach for data mining applications. Its goal is to provide a comprehensive data analytics toolbox to address challenges in manufacturing and ultimately help to establish standards for data-driven process models that can be implemented, e.g., for improving production quality, predictive maintenance, error detection, and other problems in industrial production and manufacturing.

The standard CRISP-DM framework describes a cyclical flow of the entire data mining process which contains several feedback loops. However, in many realistic data mining and modeling cases, it is an incomplete framework that requires additional information and active interactions between different sections of CRISP-DM. The demand for additional or new information can arise due to real-world production complexity, e.g., data-related or industry-specific applications, other organizational and manufacturing issues, or new emerging business trends.

Our review discussed data and model-related issues for building robust models that are successfully linked to a business understanding. One of the challenges for robust model building is the active interaction between different phases of the CRISP-DM framework. For instance, business understanding and data understanding go hand in hand because we learn business objectives (business understanding) and use relevant data (data understanding), so data should be explored appropriately (data preparation) by various statistical and analytical tools to enhance our business understanding from a realistic viewpoint. These three phases require an active cooperation by data experts and business experts so that any gap or misinformation can be quickly corrected. Similarly, each phase in the CRISP-DM framework can seek interactions through a feedback loop to clarify the process, and such interactions can be temporary or permanent extensions of the model. Thus, the active interactions between the different phases of the CRISP-DM framework should be systematically and efficiently used and managed in order to improve the whole data analysis process. Therefore, for an efficient adaption for implementing data mining and ML models one should consider a completely connected CRISP-DM, that enable an active interactions between experts to optimize a reliable and robust subclass of completely connected CRISP-DM.

We would like to note that the cyclic-nature of CRISP-DM is similar to general data science approaches (Emmert-Streib et al., 2016). Interestingly, this differs from ML or statistics that focus traditionally on a single method only for the analysis of data, establishing in this way one-step processes. Thus, CRISP-DM can be seen as an industrial realization of data science by detailing all relevant steps. However, a fully connected GCRISP-DS with active interactions is a more realistic and flexible human-centered data science model for industrial applications.

Another important issue discussed herein is related to the model assessment from the viewpoint of data experts, business experts and users. We discussed four essential components of model assessment: model accuracy, model interpretability, model multiplicity, and model transparency. All are crucial for the final deployment of the model. A model should not deviate from its goal as it encounters new data; it should predict results with accuracy and provide a satisfactory interpretation. Multiple models with competing accuracy also present a challenge when it comes to selecting a robust and interpretable model. We define cases where the interpretability of the model is crucial for business understanding and decision making.

The fourth issue is model transparency. This should allow users to obtain information on the internal structure of algorithms and methods to enable them to use various functional parameters so that the deployment phase can be implemented and integrated with the least effort and users can use the model and rely on its predictions. We suggest a documentation that includes model-related descriptions and appropriate details on business results. Specifically, we suggest the following points for maintaining transparency:

– Model performance evaluation results with simulated and surrogate data should be provided to the user. These performance evaluation should be performed considering various model and data related parameters such as: performance with imbalanced data, sample-size, correlation effect, incremental changes in data over time.
– ML Model power and false positive rate (FPR) effect on decision making should be summarized by defining a performance score with respect to business decisions. For example, How much a prediction made by a ML model could affect the business decisions in long and short run despite the models’ higher accuracy and low FPR.
– Users’ input data evaluation score comparing with training data. This information would allow users to understand the deviation of the input data from the training data characteristics.
– User should be provided with Feature importance and their stability. Various feature importance strategies discussed in Model Assessment section can be used to describe feature importance score. We must also provide business experts evaluation on importance of such features.
– Counter-intuitive predictions and features should be provided to users, if any, where business experts and data models do not agree with each other.
– Model security is one of the important concern, where the noise in the collected data from smart sensors or IoT can be crafted by a malware to give wrong decisions by a model. ML models are vulnerable of such data despite the robustness. The data and ML model should be tested and protected with such cases and it should provide warnings to users when such data is used for prediction.

## Conclusion

In this paper, we provided a review of the CRISP-DM framework and extensions thereof. A summarization of these extended models we called GCRISP-DS because it provides the most flexible and customizable framework to deal with industry-specific problems. Furthermore, we provided an in-depth discussion of model assessment and robustness-issues which are important phases of any CRISP realization.

Specifically, we reviewed the following important points for the application of data-driven knowledge discovery models:

1) Adoption of relevant and practically implementable, scalable, cost-effective, and low-risk CRISP-DM models (subclass of the GCRISP-DS framework) with industry-specific and general industrial-trend related extensions.
2) Each phase of the CRISP-DM framework should interact actively so that any data and model issue can be resolved without delay.
3) When using GCRISP-DS, define the problem category related to industrial production and maintenance for the practical implementation of data analysis. This results in a problem-specific CRISP realization (subgraph of GCRISP-DS).
4) Define the type of analysis to be performed for the model-building to avoid wrong answers to the right questions.
5) Various data-issues should be appropriately handled for data understanding and business understanding, such as exploration, preprocessing and model building (see discussion in *Model Assessment*).
6) Model evaluation and model deployment should ensure that the implemented model satisfies the requirements of model accuracy, model interpretability, model multiplicity and model transparency.
7) The GCRISP-DS framework adopts a human-centric approach to allow for transparent use of the model so that users and developers can evaluate, customize, and reuse the results that enhance their understanding of better production quality, maintenance, and other production, business, and societal goals.

In general, for data-driven models, data are at the center stage and all goals are realized by the potential of the available data (Emmert-Streib and Dehmer, 2019a). The assessment of achieved business goals at the end-phase of a CRISP realization is also given through the success of the model. Thus, all novel understanding depends on the data obtained and how we turn such data into useful information through systematic and collaborative means. These objectives cannot be obtained solely through the efforts of business experts or data experts. Instead, a collective effort of active cooperation is required to establish a human-centered GCRISP-DS for achieving the business goals. Such an interaction forms a complex system that leads to the emergence of various higher-level characteristics of KDD models in smart manufacturing. In such a complex system, the various AI and ML tasks must be transparent, interactive, and flexible in order to create a robust and stable manufacturing and production environment. Future research in this direction will explore industry-specific requirements to develop data-driven knowledge discovery models and their implementation in practice.

The future challenges of GCRISP-DS are to develop methods and frameworks that could effectively estimate and measure the interactions and their effect between different phases considering various influential factors in and between the phases. For example, by quantifying these with with a quality score considering various data-related features such as sample-size, variance, data dimensionality, missing-values, and external effects. One could optimize the requirements to be fulfilled by each phase considering data quality scores and model performance scores for implementing a cost-effective and robust model. Similarly, different phases contribute to the cost and robustness depending on various other factors: domain-related challenges, technical challenges, and model implementation challenges. Overall, such factors provide significant obstacles for a robust implementation of GCRISP-DS.
